# The Edible Plant Microbiome represents a diverse genetic reservoir with functional potential in the human host

**DOI:** 10.1038/s41598-021-03334-4

**Published:** 2021-12-15

**Authors:** Maria J. Soto-Giron, Ji-Nu Kim, Eric Schott, Claudine Tahmin, Thomas Ishoey, Tracy J. Mincer, Jillian DeWalt, Gerardo Toledo

**Affiliations:** 1 Solarea Bio, 100 Edwin H Land Blvd, Cambridge, MA 02142 USA; 2 Department of Biology, Wilkes Honors College, Jupiter, FL 33458 USA; 3 Present Address: Harbor Branch Oceanographic Institute, Florida Atlantic University, Fort Pierce, FL 34946 USA

**Keywords:** Food microbiology, Metagenomics, Microbial ecology, Microbiome, Nutrition

## Abstract

Plant microbiomes have been extensively studied for their agricultural relevance on growth promotion and pathogenesis, but little is known about their role as part of the diet when fresh fruits and vegetables are consumed raw. Most studies describing these communities are based on 16S rRNA gene amplicon surveys, limiting our understanding of the taxonomic resolution at the species level and functional capabilities. In this study, we characterized microbes colonizing tomatoes, spinach, brined olives, and dried figs using shotgun metagenomics. We recovered metagenome-assembled genomes of novel lactic acid bacteria from green olives and identified high intra- and inter-specific diversity of *Pseudomonas* in tomatoes. All samples were colonized by *Pseudomonas,* consistent with other reports with distinct community structure. Functional characterization showed the presence of enzymes involved in vitamin and short chain fatty acid metabolism and degradation of diverse carbohydrate substrates including plant fibers. The dominant bacterial members were isolated, sequenced, and mapped to its metagenome confirming their identity and indicating the microbiota is culturable. Our results reveal high genetic diversity, previously uncultured genera, and specific functions reflecting a likely plant host association. This study highlights the potential that plant microbes can play when consumed as part of our diet and proposes these as transient contributors to the gut microbiome.

## Introduction

Advancements in molecular methods over the past 30 years have expanded our knowledge of the vast extent of biological diversity on earth. The use of 16S rRNA gene sequences has enabled characterization of microbial communities in a wide range of habitats including the human body and in fluids including stool, prompting establishment of the microbiome field and industry. Diet has been identified as one of the main variables shaping the gut microbiome, as dietary intervention studies have demonstrated that the gut microbiota composition can reflect variations in food consumption despite immense inter-individual variation and the heterogeneous nature of stool that complicates representative sample collection^1–3^.

In contrast to the efforts aimed to describe the microbiota in stool, very little exploration has been conducted in our foods and the microbes consumed in a typical diet^4^. Most of what we know about food microbiomes, especially as it relates to fresh fruits and vegetables, is related to agricultural pathogens and toxins. However, the recognition that our diets contain potentially beneficial bacteria and fungi is a new concept. Berg et al.^5^ coined the term *Edible Plant Microbiome* to identify the types of tissues, compartments, and plant groups that carry live microbes in our diets, especially when eaten raw. The overall view is that microbes colonizing internal and external plant tissues can play a role in human nutrition and health and provide insights of co-adaptation processes between plants, animals, and their microbiomes. For example, Wasserman et al.^6^ studied the microbiomes of cruciferous vegetables and showed they provide protection against fungal plant pathogens and anticancer molecules to the human host, indicating an important relationship between the plant host and its microbiota. Plant-associated bacteria play crucial roles in their host, including beneficial effects on the production of secondary plant metabolites, protection against pathogen colonization, plant growth promotion, among others^7,8^. However, some metabolites such as plant hormones produced in concert with the plant microbiome can also be relevant in the human gut^9^, suggesting a co-adaptation to colonize both the plant and animal host.

The microbial diversity of fresh fruits and vegetables commonly eaten raw has mainly been studied using both 16S rRNA gene sequences and cultivation approaches. For example, baby spinach and romaine, green leaf, iceberg, and red leaf lettuce^10–13^, cruciferous vegetables^6,13^, tomatoes^14,15^, and fruits such as peaches, strawberries, grapes^13^, bananas^16^, and apples^17^ have all been studied by this approach. These studies have in most cases reported a microbial community dominated by Gammaproteobacteria, followed by Bacteriodetes, Actinobacteria, and Firmicutes. Kõiv et al.^18^ analyzed five tubers (potato, carrot, beet, neep, and Jerusalem artichoke) by separating the peels from the pulp and found the peels were enriched in Alphaproteobacteria and Actinobacteria, while the pulps were enriched in Gammaproteobacteria and Firmicutes. At the genus level, *Pseudomonas* seems to be the most common and abundant taxon across samples in most studies, indicating it is consumed in raw items that are considered part of a healthy diet. However, the extent of intraspecific genetic diversity and possible roles in its passage through the gastrointestinal (GI) tract are largely unknown. The abundance of viable bacteria and fungi living in leafy salad vegetables ranges from 8.0 × 10^3^ to 5.5 × 10^8^ CFU/g^12^ and the bacterial fraction, based on 16S rRNA gene copy numbers in one organic apple, averages 4.85 × 10^7^ 16S rRNA gene copy numbers per gram^17^. Tubers carry from 1 × 10^3^ to 5.5 × 10^8^ CFU/g in their pulps^18^. The consumption of raw fruits and vegetables in salads is considered healthy and provides abundant and diverse microorganisms that may be functionally active along the passage through the GI tract of the consumer.

Fermented foods are another dietary component rich in microbes. They were consumed more frequently in the past as pickling and fermenting were means to preserve and extend food shelf life prior to the use of chemical preservatives and refrigeration. They carry endogenous communities of Lactic Acid Bacteria (LAB) that become enriched during the fermentation process. For example, brined olives carry significant amounts of *Lactobacillus* suggesting a possible probiotic role in olives^19,20^. Another class of fermented foods is represented by cheeses and other dairy products. Passoli et al.^21^ analyzed 303 metagenomes from different fermented foods, cheese, yogurt, and dietary supplements and recovered Metagenome-Assembled Genomes (MAGs). Authors compared these MAGs to reference genomes and MAGs from human stool and established a link between some microbial members present in foods that can be shaping the gut microbiome.

Shotgun metagenomic approaches are required to characterize the microbial community at higher resolution including strain and functional gene levels. Cernava et al.^22^ analyzed the arugula phyllosphere and reported the resistome as part of the gene content with ecological significance. These types of analyses are useful to describe the microbial diversity and the linkage with metabolic pathways associated with plant fiber degradation, vitamin biosynthesis, and short chain fatty acid metabolism in plant-associated microbiomes.

In this pilot study we developed methods to extract microbial cells including the endophytic communities from plant tissues and sequenced them extensively to generate a shotgun metagenomic dataset from tomatoes, baby spinach, green olives, and dry figs. We observed a high degree of intraspecific diversity and possibly novel genera in the samples when MAGs were recovered and analyzed. In addition, we isolated the dominant bacterial species of each sample and sequenced their genomes. Our results suggest the Edible Plant Microbiome is a functional component of the diet that might contribute to the gut microbiome with potential implications for human nutrition and health.

## Results

### Microbial community structure of the edible plant microbiota

We focused our study on fruits and vegetables that are part of a common diet, easily accessible to consumers, and commonly consumed raw; therefore carrying viable microbes. The samples for this study were acquired from supermarkets and prepared in a similar way for human consumption by rinsing vigorously with tap water. The samples were then homogenized by blending, and the plant-associated microbiome was purified from plant cells by filtration, centrifugation, and the use of detergents to preferentially lyse plant cells and chloroplasts.

DNA was extracted from the purified microbiomes and analyzed by shotgun metagenomic sequencing. Plant cell removal efficiency was verified by fluorescence microscopy using DNA stains, indicating ranges between 70 and 95% efficacy, but in all samples, plant cells and DNA remained present in the shotgun metagenomic datasets (ranging from 0.1 to 69.3% of the total number of sequencing reads) (Supplementary Table 1).

**Figure 1:**
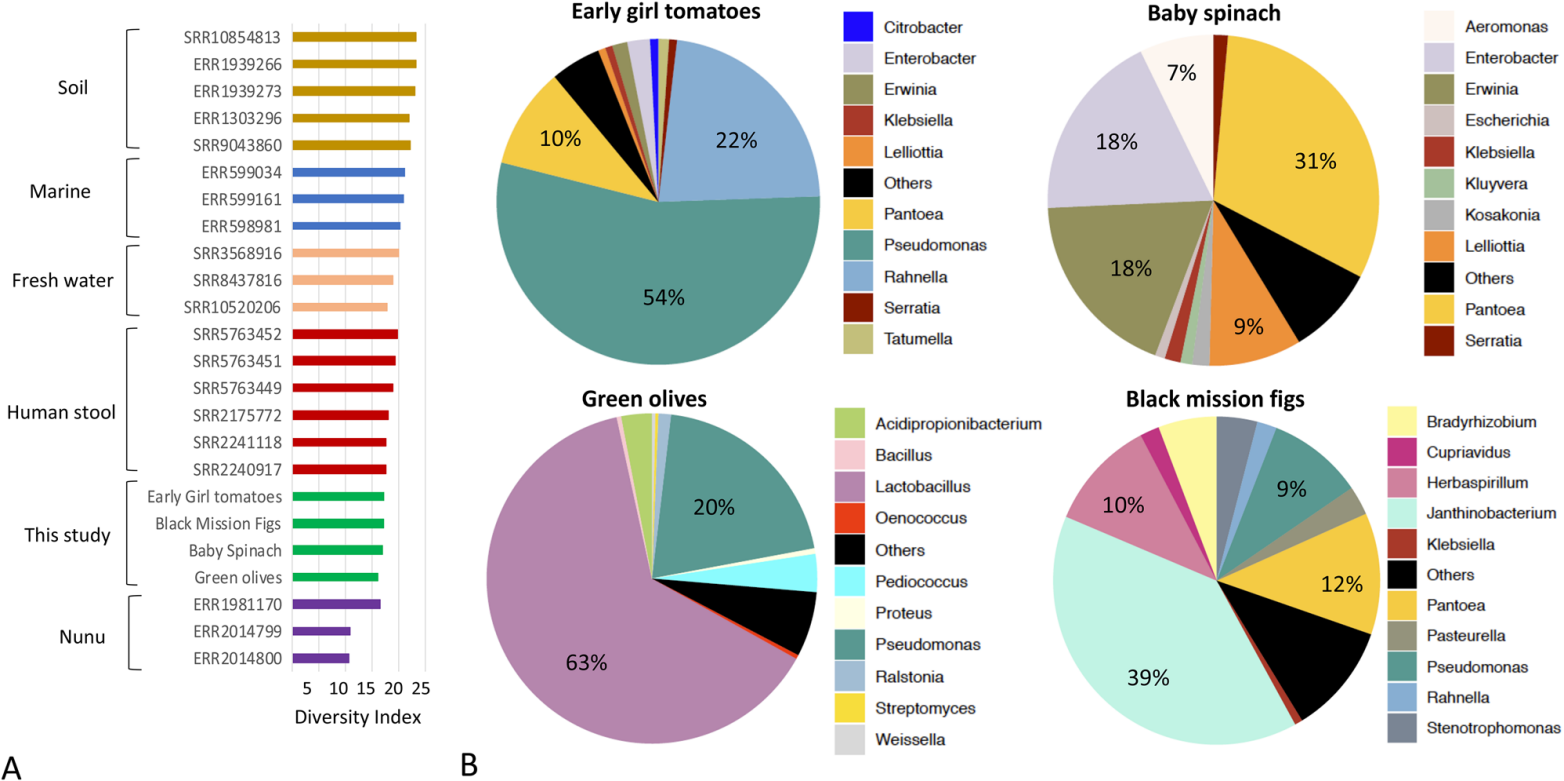
Diversity and taxonomic composition of shotgun metagenomes from fruits and vegetables analyzed in this study. (**A**) Comparison of alpha diversity index values of metagenomic samples from this study with other metagenomes from diverse ecosystems. Diversity index values were calculated based on the rarefied coverage of metagenomic reads in each sample using Nonpareil v3^23^. Bars are colored by sample type and each sample is labeled by its accession number submitted at the NCBI Sequence Read Archive (SRA) or the EBI European Nucleotide Archive (ENA). Metadata associated with these metagenomic samples can be found in Supplementary Table 2. (**B**) Pie charts showing the relative abundance of the top ten most abundant microbial members at the genus level based on k-mer analysis using kraken2^24^.

After host DNA removal, shotgun metagenomic samples ranged from 3.6 to 23 million sequencing reads with an average read length of 100 bp. The coverage estimation of the microbial community based on read redundancy indicated that more than 80% of the communities were covered in the samples, suggesting our sequencing effort captured the majority of abundant microbes present in the sample (> 0.1% abundance of the total) (Supplementary Fig. 1). Comparison of sequence diversity based on metagenomic reads (similar to alpha diversity obtained from Nonpareil curves) with other metagenomes from different environments showed that samples from this study present similar diversity to the ones seen in the human stool and slightly higher than Nunu, a west African fermented yogurt-like milk product, but lower than marine, fresh water, and soil samples (Fig. 1A).

Taxonomic annotation based on metagenomic reads indicated that the microbial communities were dominated by Proteobacteria in all samples, except for green olives in which the most abundant phylum was Firmicutes (69.7% of the total). Actinobacteria were also 10 × more abundant in green olives compared to the other samples (4.2% vs. 0.4% on average). At the genus level, vegetables and fruits were dominated by different microbial members (Fig. 1B). For instance, *Pseudomonas* was the most abundant taxon in early girl tomatoes (54%) followed by *Rahnella* (22%) and *Pantoea* (9%). Baby spinach were dominated by *Pantoea* (30%) followed by *Erwinia* (20%) and *Enterobacter* (19%). Black mission figs were dominated by *Janthinobacterium* (38%), followed by *Pantoea* (12%) and *Herbaspirillum* (12%). Green olives were dominated by *Lactobacillus* (62%) followed by *Pseudomonas* (21%), A*cidipropionibacterium* (3%), and *Pediococcus* (3%). The microbial composition in these samples is consistent with previous reports on Gammaproteobacteria as a relevant group in plant-associated microbiomes and the genus *Pseudomonas*^10–12,22^, a dominant member across samples*.* However, further comparisons between samples would require additional sampling as microbiomes can be variable in response to multiple factors such as farming practices.

The taxonomic profile based on 16S and 18S rRNA gene sequences recovered from the metagenomes showed that black mission figs harbored the highest proportion of sequencing reads belonging to fungi, mainly classified in the *Aspergillus* (30%), Unclassified Eurotiales (29%), and Unclassified Trichocomaceae (26%) genera (Supplementary Table 3). No archaeal sequences were detected in any of the metagenomes.

**Figure 2:**
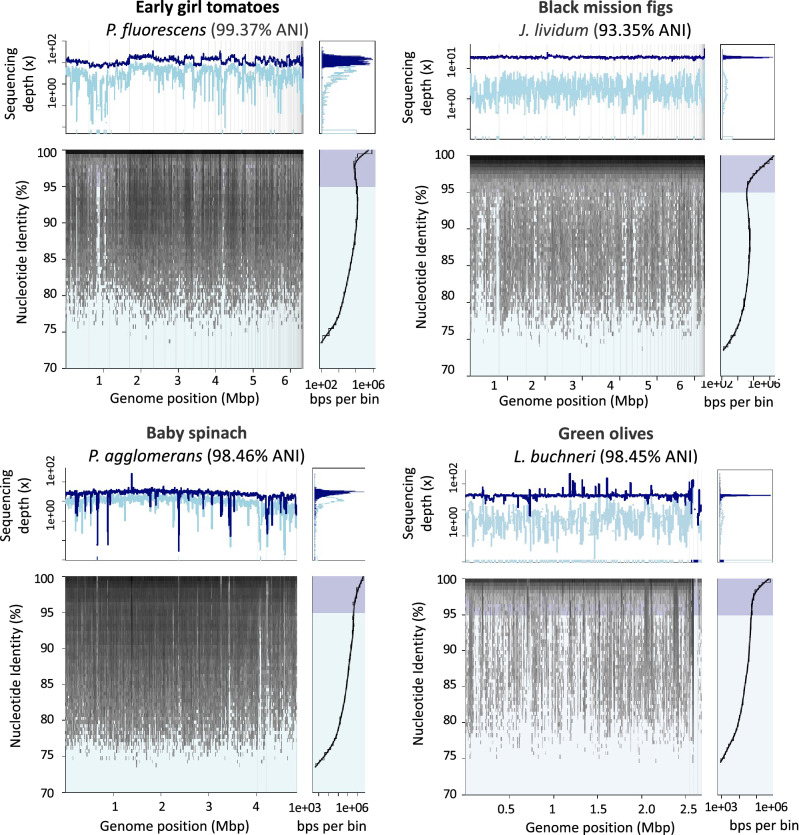
Fragment recruitment plots of bacterial isolates derived from fruits and vegetables and fermented foods against metagenomic sequencing reads from each sample. The y-axis in the top left panel corresponds to the average sequencing depth values in logarithm scale of reads that mapped to the genome sequence (x-axis). The y-axis in the bottom left panel corresponds to the percentage of nucleotide identity (%) of each mapped read and the x-axis to the position of the read on the genome. The right bottom panel shows the identity histogram of mapped reads (logarithmic scale). The dark blue peak in the histogram (right top panel) corresponds to the average coverage of mapped reads with ≥ 95% nucleotide identity, while the light blue indicates the coverage of sequencing reads with < 95% nucleotide identity. A sequence-discrete population is represented by reads with high nucleotide identity (> 95% nucleotide identity, indicates the cut-off for species demarcation) to the reference genome sequence and with even coverage across the reference sequence.

We also generated a strain culture collection from these samples using different media types and identified representative species from multiple genera that were present in high abundance in the metagenomes. For instance, bacterial isolates from *Pseudomonas*, *Rahnella*, and *Pantoea* were isolated from early girl tomatoes; *Pseudomonas*, *Pantoea*, and *Stenotrophomonas* were isolated from baby spinach; *Lactobacillus*, *Pseudomonas*, *Bacillus*, *Leuconostoc*, and *Pantoea* from green olives; and *Janthinobacterium*, *Herbaspirillum*, *Pantoea*, and *Pseudomonas* from black mission figs. Most of the isolates belong to known species represented in public databases except for one isolate from figs (*J. lividum*, 93.35% ANI), which likely represents a new species. Fragment recruitment plots of the genome of some of these bacterial isolates against the metagenomic reads for each sample showed the presence of the isolate in the sample (nucleotide identity > 99%) in high abundance. For instance, the average sequencing depth of *P. fluorescens* from early girl tomatoes was 12.7 × while the one for *J. lividum* from black mission figs was 53.9 ×. In addition, to the target genome, there is a gradient of identities that reveal possible ecotypes (Fig. 2).

To identify individual uncultured populations that represent abundant members in the microbial community, genome binning analysis was performed by assembly of sequencing reads into contigs and then binning of these contigs into metagenome-assembled genomes (MAGs) (Supplementary Table 4). Quality of MAGs was evaluated following the standard criteria reported by Bowers et al.^25^. MAGs with more than 90% completeness and less than 5% contamination were classified as high quality, while MAGs with > 50% completeness and less than 10% contamination were classified as medium quality. In total, 12 MAGs (8 high and 4 medium quality) were recovered from the metagenomes with identities matching dominant genera seen by Kraken2^24^.

The majority of recovered MAGs were obtained from green olives (n = 7), likely because of the higher average coverage of the microbial community (Nonpareil coverage: > 95%) and low complexity of the community (*Lactobacillus* and *Pseudomonas* encompass approx. 82% of the total abundance). Five near-complete MAGs were affiliated with lactic acid bacteria confirming the dominance of this group in green olives with high coverage for each. More specifically, MAG3 showed 99.7% whole-genome average nucleotide identity (ANI)^26^ to *Lactobacillus buchneri*. MAG9 showed 98.8% ANI to *Lactobacillus acetotolerans,* but MAG1, MAG10, and MAG6 could not be assigned to a known species following the 95% ANI threshold for species demarcation or to a genus (> 80% ANI), most likely representing undescribed microbial members^26^. These MAGs can only be assigned to the family Lactobacillaceae. One of the two medium quality MAGs was assigned to the genus *Pseudomonas* and the other one to Gammaproteobacteria.

Two medium quality MAGs from the metagenomic sample taken from baby spinach were annotated as *Erwinia persicina* (93.4% ANI) and *Aeromonas* sp. (97.7% ANI). In black mission figs, three high quality MAGs were recovered affiliated with *Janthinobacterium* (93% ANI), *Pantoea* (91% ANI), and *Herbaspirillum* (87.5% ANI) genera, most likely representing undescribed bacterial species given their nucleotide divergence relative to known species. These MAGs are consistent with the taxonomic description of the samples based on Kraken2.

### Isolation of representative bacterial strains from each sample and genome sequencing

In order to evaluate if the bacterial diversity in the samples was amenable to culture, strains were isolated using different formulations of solid media and selective conditions. In addition, colony forming units (CFU) were counted on the microbial preparations showing counts between 1 × 10^4^ and 1 × 10^6^ CFU/g consistent with other studies. The most abundant species on each sample was isolated and its genome was sequenced. Fragment recruitment plots of the dominant genome of each sample against the metagenome from where it originated revealed high relative abundance in the sample, high sequence identity to the recruited genome, and in most cases a high degree of intraspecific diversity (Fig. 2). Some of the isolates from green olives matched the MAGs reconstructed from the metagenome as in the case of MAG3 and the isolate *L. buchneri*.

### Diversity of *Pseudomonas* populations across samples

**Figure 3:**
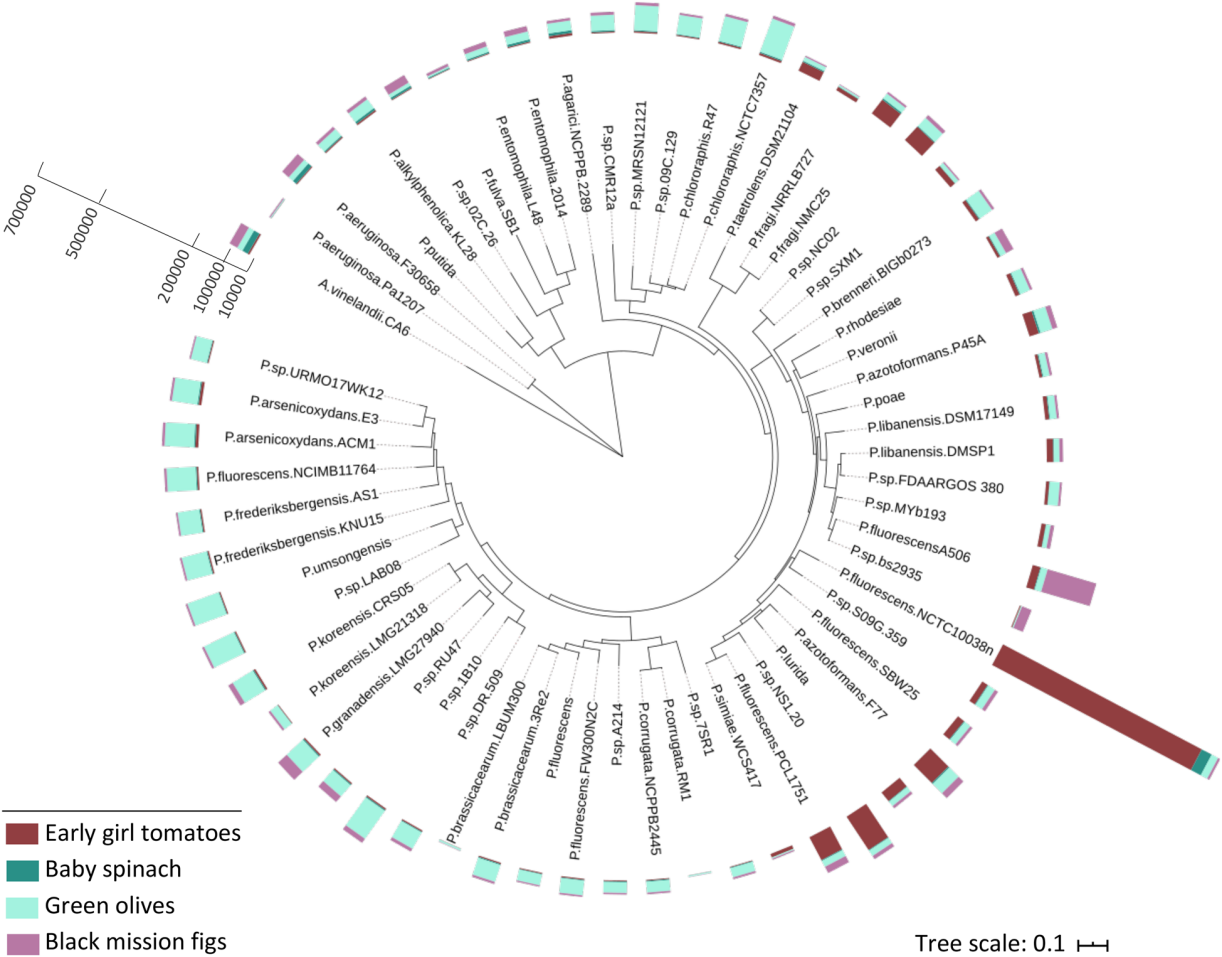
*Pseudomonas* population diversity in fresh fruits and vegetables. Maximum-likelihood tree constructed using 100 universal single copy genes from 26 *Pseudomonas* reference genomes. *Azotobacter vinelandii* was used as an outgroup. Bar plots colored by sample type show the number of sequencing reads that mapped each genome. Phylogenetic tree was visualized and edited using iTOL v6 (https://itol.embl.de/)^27^.

The distribution of metagenomic reads mapped onto a phylogenetic tree of *Pseudomonas* constructed with 100 marker genes from representatives of different species revealed the presence of multiple *Pseudomonas* species across samples as well as different distributions of these species per sample (Fig. 3).

Due to the elevated genetic diversity previously observed in *P. fluorescens* in soil and other habitats/environments, its importance as a plant growth-promoting bacterium, and presence and abundance across samples, we were interested in evaluating the level of intraspecific population diversity of this taxon in food ingredients that are part of our diet (Supplementary Fig. 2). Here, the genome from the reference strain *P. fluorescens* NCTC 10038 was used to recruit metagenomic reads and visualize the nucleotide diversity of *Pseudomonas* populations in the samples. Fragment recruitment plots showed that early girl tomatoes contained high-identity nucleotide sequencing reads evenly across the entire reference genome, most likely belonging to members of the same species (> 95% nucleotide identity and 12X of average sequencing depth). In addition, diverse co-occurring populations seem to be present as well (mapped reads with 80% to 95% nucleotide identity), indicating other species in the same genus. Baby spinach also showed reads mapping to the reference genome, but in less abundance (0.61X coverage) and lower species diversity. *Pseudomonas* was not one of the top 10 genera in this sample but the *P. fluorescens* NCTC 10038 genome could be recovered. Metagenomes from green olives and figs do not contain *P. fluorescens* NCTC 10038 but display more distantly related populations present in the sample (nucleotide identity ranging from 80 to 95%).

### Genomic diversity and functional profile of LAB-like MAGs recovered from green olives

Since five MAGs belonging to LAB from green olives were recovered, we were interested in evaluating their genome diversification and function which can reflect ecological niches in the community. For this, metagenomic reads were recruited against the MAG sequences in the sample (Supplementary Fig. 3). The resulting fragment recruitment plots showed that LAB-like MAG6 and MAG3 harbored considerable intraspecific diversity (mapped metagenomic reads with nucleotide identity values between 95 and 100%), and that moderately-to-distantly related populations (reads with < 90% identity) are co-occurring in green olives. In contrast, recruitment of reads by the LAB-like MAG1 and MAG10 indicated one dominant population (48.7X and 47.2X mean sequencing depth, respectively) with less population sequence diversity (e.g., few reads mapping > 95% and 99% nucleotide identity to MAG sequence). The conditions of pickled green olives favor the dominance of multiple LAB species, some with high genetic diversity and others distantly related to known species and likely representing a novel genus.

**Figure 4:**
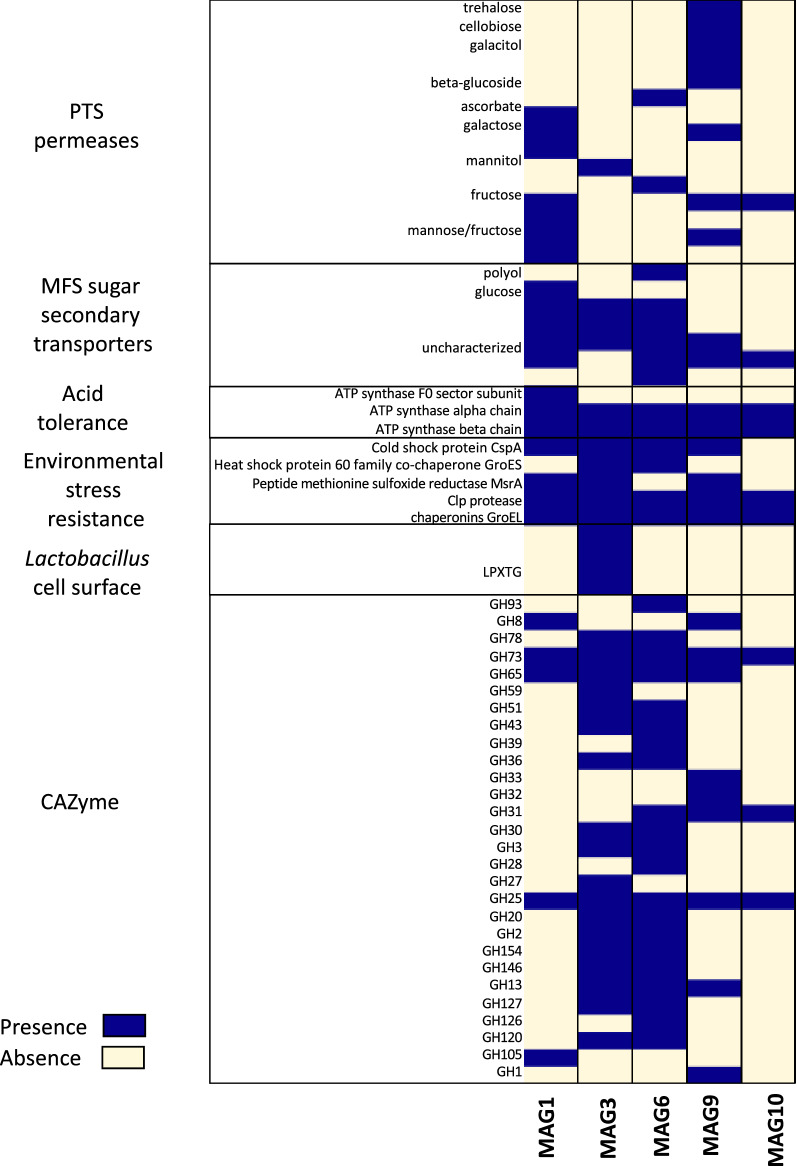
Functional profile of LAB-like MAGs recovered from the green olives metagenome. Functional properties identified in the LAB-like MAGs that have been previously reported in commercial probiotic strains^30,31^. These proteins include Phosphotransferase Transporter System (PTS) permeases, secondary sugar transporters from the major facilitator superfamily (MFS), LPXTG cell wall anchor domain-containing protein, among others.

LAB, in particular, *Lactobacillus*, are some of the best characterized and widely studied probiotic bacteria, and have been isolated from sources such as stool, milk, and soil. Their probiotic attributes include antimicrobial activities, immunomodulatory cell wall components, adhesive pili, and enzymes for SCFA production^20,28,29^. We screened for genes with probiotic-linked mechanisms in LAB-like MAGs with functions that are related with host colonization and anti-inflammatory properties (Fig. 4).

The LAB-like MAGs showed the presence of MFS sugar secondary transporters, Phosphotransferase Transporter System (PTS) permease families, and anchor proteins including LPXTG cell wall anchor domain-containing proteins that have previously been involved in conferring beneficial effects to the host. PTS permeases in *Lactobacillus* are associated with fermentation of various sugar substrates^32^. In addition, PTS permeases have been associated with the long-persistence phenotype in the gastrointestinal tract observed in the probiotic *L. johnsonii* NCC533^33^. LPXTG cell wall anchor domain-containing proteins have been widely studied in *Lactobacillus* because of their role in epithelial cell adhesion in the gastrointestinal tract and in host immune response modulation^29,34^. Additional beneficial features associated with probiotics are tolerance to environmental stress, including acidity**.** The gene coding for the heat shock protein 60 family co-chaperone GroEL that confers environmental stress resistance was identified in the MAGs, which allows *Lactobacillus johnsonii* to bind to mucin and intestinal epithelial cells to enhance intestinal persistence^35^. Altogether, these data suggest that green olives and their microbes can offer some of the same functionalities provided by commercial probiotics.

### Functional annotation of the edible plant microbiota

Beyond taxonomic annotation, shotgun metagenomic sequencing facilitates extensive analysis of all functional genes present in a microbial community. With this, we can begin to better understand not only who is present, but what functions they can perform. Here, functional annotation of genes from the metagenomes using UniProt DB^36^ revealed that the majority of the genes predicted in the sample from early girl tomatoes, baby spinach, and green olives were annotated (> 90% of the total genes), while only around 65% of the genes predicted in the sample from figs were annotated (Table 1). The lower annotation of the genes in figs can be explained by a large fungal population (10%) and the paucity in fungal databases and genomes. Prediction of Gene Ontology (GO) biological processes showed that a significant proportion of metagenomic reads were related to housekeeping functions such as nucleic acid metabolism, protein metabolism, and carbohydrate metabolism (Supplementary Table 5).

**Table 1:** Functional annotation of predicted genes from metagenomics samples using carbohydrate-active enzymes (CAZyme) and UniProt databases.

No. of annotated genes	Early girl tomatoes	Baby spinach	Green olives	Black mission figs
	841	1011	881	748
AA (%)	3.1	2.0	1.2	6.1
CBM (%)	5.0	5.4	2.3	3.6
CE (%)	5.4	3.7	4.7	9.1
GH (%)	43.2	46.9	56.9	38.6
GT (%)	40.9	39.8	34.2	40.6
PL (%)	2.5	2.3	0.8	1.9

Columns indicate the number of predicted proteins, percentage of each CAZyme module.

*AA* auxiliary activity, *CBM* carbohydrate binding module, *CE* carbohydrate esterase, *GH* glycoside hydrolase, *GT* glycosyltransferase, *PL* polysaccharide lyase, and the percentage of annotated proteins using UniProt database^37^.

Comparison of the annotated genes across the four samples revealed that not all biological processes are equally distributed. In baby spinach, we identified a high frequency of reads assigned to processes including the pectin catabolism, glycerol ether metabolism, trehalose biosynthesis, L-threonine catabolism to glycine, and L-serine biosynthetic process compared to the other samples. In figs, we found that the xyloglucan metabolism and the poly-hydroxybutyrate biosynthesis were more frequent. In tomatoes, glyoxylate catabolism was more frequent in comparison with the other samples.

We also identified the presence of metabolic pathways associated with human gut microbiome homeostasis including biosynthesis of glutamate, terpenoids, riboflavin, thiamine, folic acid, glutamine, and biotin. Carotenoid biosynthetic process was present in all samples, except in green olives, while response to redox state, lactose metabolic process, as well as lipoteichoic acid, teichoic acid, and isopentenyl diphosphate biosynthetic process was identified only in green olives. Overall, these data revealed the Edible Plant Microbiome harbors a diverse genomic repertoire that varies among samples.

### Diversity of carbohydrate-active enzymes

To further understand the functional capacity of these plant microbiomes, we investigated the abundance of carbohydrate-active enzymes (CAZymes), a group of enzymes that are involved in the breakdown of plant cell walls with potential for further conversion to beneficial metabolites in the human intestinal tract. CAZymes are categorized based on protein sequence and structural similarities including glycosyltransferases (GTs), glycoside hydrolases (GHs), carbohydrate esterases (CEs), polysaccharide lyases (PLs), auxiliary activities (AA), and carbohydrate-binding modules (CBMs). Putative CAZymes in the plant-associated microbiota characterized in this study were identified using predicted open reading frames (ORFs) from the metagenomes and the CAZymes database^38^. GHs and GTs were the most abundant CAZymes predicted in the metagenomes (approx. 30 to 57% of the total). Fewer percentages of AAs (approx. 1–6%), CBMs (2–5%), CEs (3–9%), and PLs (1–4%) enzymes were identified in the samples (Table 1 and Supplementary Table 6).

Specifically, 79 of the 133 GHs families in the CAZy database were detected among samples. GHs are enzymes that hydrolyze the glycosidic bond leading to the formation of carbohydrate hemiacetal^38^. Our results showed common GHs present in all metagenomes with GH13, GH23, and GH1 being the most frequent families. GH13 is one of the most common families identified in the human gut microbiome and is characterized for breaking down starch^39^. Starch is the major alpha-glucose polymer that plants synthetize to store energy and an important carbohydrate source consumed worldwide^40^. We also observed GHs that were distinctly distributed in specific samples and that have been associated with beneficial effects in the human gut. For example, the enzyme tomatinase (EC 3.2.1.-) is a member of GH10 along with xylanases. Tomatinase facilitates the degradation of the antifungal tomatine into nonfungitoxic substrates: tomatidine and beta-lycotetraose. The presence of fungi in black mission figs may be linked to the presence of GH10 enzymes rendering a fungal toxin inert.

GH31 was prevalent in green olives and baby spinach. The enzyme sulfoquinovosidase (EC 3.2.1.) in GH31 hydrolyses alpha-sulfoquinovoside. Sulfoquinovose is produced by plants, degraded by aerobic bacteria into carbon and sulfur^41^, and present in leafy green vegetables such as spinach^42^. Sulfoquinovosidase has also been implicated in benefitting human gut commensals^41^. GH1 comprises B-glucosidases involved in cellulose degradation, one of the most abundant fibers in leafy vegetables. GH1 enzymes are used by gut microbes to hydrolyze glycans into glucose and glycolytic precursor glucose-6-phosphate. GH65 containing trehalose phosphorylases was the most abundant in the green olive sample. Trehalose is a disaccharide which prevents desiccation in plants and is present in olive leaves^43^. Trehalase is also present in the small intestine of vertebrates, and bacteria such as *Enterococcus* have been shown to metabolize trehalose. The family GH28 contains enzymes related to pectin degradation. Pectin is considered a prebiotic, and bacteria belonging to the genera *Erwinia* and *Pseudomonas*, present in tomatoes, are common pectinolytic enzyme producers^44^. GH28 enzymes produced by bacteria have been found to be active in the oral cavity commencing the breakdown of pectin when ingested^45^. Taken together, these data further support the notion that the microbes present in plants could provide metabolic benefit to humans when consumed as part of the diet, as they contain a large number of carbohydrate active enzymes that play crucial roles in human health and the substrates which are present in plant cell walls.

### Metabolic pathways associated with SCFAs production

The most widely studied microbial metabolites in relation to human health are short chain fatty acids (SCFAs). SCFAs are derived from the microbial fermentation of complex carbohydrates, and act as both important regulatory signaling molecules and an additional fuel source for the host^46,47^. The most prevalent SCFA pathways across the four samples analyzed were lactate oxidation and L-lysine catabolic process to acetate (Supplementary Fig. 4A). Lactate metabolic process was also identified in the metagenomes of both baby spinach and green olives. The least common pathways represented across the metagenomes were butyrate metabolic processes, as the metagenomes contained the fewest reads mapping to butyrate kinase. Interestingly, the sample with the broadest SCFA metabolic potential was the pickled green olive, suggesting the pickling process may select for microbes with extensive SCFA productive capacity. In agreement with Supplementary Fig. 4A, the enzymes with the greatest representation across samples are those related to acetate (acetate kinase) and lactate (d-lactate dehydrogenase) production. These data indicate that the microbiomes of fruit and vegetables contain the genes and pathways required to produce acetate and propionate, a family of microbial metabolites associated with human health, but are missing butyrate biosynthesis as it is mostly linked to strict anaerobes not seen in the samples.

### Biosynthetic gene pathways associated with vitamin production

Microbes are known to synthetize vitamins, which are essential for several metabolic reactions in their respective hosts. The identification of enzymes that participate in vitamin biosynthesis and uptake transporters in the metagenomes of this study was based on genes annotated to KEGG Orthologies (KOs) predicted as previously identified in human fecal metagenomes^48^ and in the probiotic strain *Bacillus clausii* ENTPro^30^ (Supplementary Fig. 4B).

Our results showed the presence of genes encoding enzymes in the plant-associated metagenomes that are part of biosynthetic pathways for cobalamin, biotin, and pantothenate. Few clusters of genes were observed for folate, menaquinone, and niacin biosynthesis. Moreover, a high proportion of genes (33/66) associated with cobalamin metabolism was detected in the metagenomes and a slightly higher abundance of cobalamin metabolic genes was found in early girl tomatoes and green olives (Supplementary Fig. 4B). Cobalamin (vitamin B12) is an essential cofactor for humans synthetized by some bacteria and archaea and it has been involved in mediating microbe-microbe interactions and host-microbe communication^49^. Cobalamin deficiency can result in anemia, nervous system disease, and neuropathy^50^.

## Discussion

The presence and the importance of microbes in food have been previously studied, suggesting that the microbes we consume in our diet can modulate the gut microbial structure. Based on these findings, together with the Edible Plant Microbiome concept previously described by Berg et al.^5^, we were interested in characterizing the diversity and functional capabilities of the Edible Plant Microbiome in common dietary fruits and vegetables. In this pilot study, we showed that microbes residing in fresh fruits and vegetables harbor a great deal of taxonomic and functional diversity that could potentially play a previously unappreciated role in shaping the human gut microbiota with downstream implications for human health. Our study was made possible by developing plant cell removal/filtration methods and performing shotgun metagenomic sequencing to provide enough sequencing coverage of the microbial communities (Supplementary Fig. 1).

Consistent with previous studies of plant associated microbiomes, Proteobacteria, Firmicutes, and Bacteroidetes were the most abundant phyla across samples (Fig. 1B). At the genus level, we observed that each sample harbored a different community structure, which may be driven by adaptations to distinct environmental factors including farming, and processing (e.g., figs were dried, and olives were pickled). We observed that several *Pseudomonas* species were present in all samples, confirming its ecological adaptation to colonize multiple crops and diverse metabolic capacity to use different organic compounds as carbon sources^51^. Specifically, *P. fluorescens* has been recognized as a bacterial complex with strains assigned to more than 50 species due to its elevated genetic diversity and gene content differences^52^. Although the genomic diversity of this taxon has been widely studied in soil and plants because of its relevance as a biocontrol agent and plant growth properties, little is known about its role and the extent of diversity in daily food items.

Fragment recruitment plots of the *P. fluorescens* reference genome against metagenomic reads from early girl tomatoes and baby spinach revealed the presence of an abundant population as well as a gradient of nucleotide diversity, most likely representing multiple discrete populations. In the case of early girl tomatoes, we observed one abundant genome population co-occurring with populations from other species, reflecting genomic differences associated with distinct ecological roles (Supplementary Fig. 2). This result echoes previous reports in other environments such as soil and lakes where microbial communities harbor discrete, but still related, populations^53–55^.

This genomic diversity can represent different ecotypes adapted to changes in environmental conditions through the plant lifecycle, soil environment, farming practices, and post-harvest handling. Therefore, each time we eat fresh fruits and vegetables we consume a variety of *Pseudomonas* species. Tracing studies will reveal their survival through the gut and potential role in the human microbiome. In addition, the poor representation of plant-associated microbiomes and the updating of new species in public databases constrain our current understanding of the ecological role of these microbes in the human gut microbiota. MAGs belonging to *Pseudomonas* have been previously recovered from gut metagenomes of human populations around the world^56,57^, but it remains unknown whether they are commensals or transient members in the GI tract.

Green olives are part of the Mediterranean diet and are widely consumed around the world. They have been recognized as an important food with respect to human health as they are rich in phenolic compounds, fatty acids, and antioxidant properties^58,59^. Moreover, during the pickling process, green olives are enriched by LAB, mainly *Lactobacillus* populations, many of which (i.e., *L. plantarum*, *L. pentosaceus*) have been recognized as probiotics due to their health benefits in humans^19,20^. By applying binning analysis, we were able to recover five near-complete MAGs that were closely related to *L. buchneri* and *L. acetotolerans*, 99.7% and 98.2% ANI respectively, as well as MAGs that might represent novel genera within the Lactobacillaceae family based on the ANI metrics for genus definition (> 80% ANI) (Supplementary Table 4). Fragment recruitment plots of these MAGs against the metagenomic reads showed a high degree of nucleotide diversity of LAB populations that are co-habiting in pickled green olives. This taxonomic diversity reflects the diverse functional profile observed in gene content differences among MAGs such as genes involved in sugar uptake, stress response, adhesion mechanisms as well as distinct CAZyme families targeting multiple substrates. This genomic diversity might be associated with the ability to colonize different niches in the plant, survive during the pickling process, and tolerate the human stomach pH to reach different compartments of the GI tract. Still, it remains unexplored the impact that the microbial diversity observed in green olives can have during its transit in the GI tract as well as in the resident *Lactobacillus* populations, that represent on average 0.3% in the colon^60^ and 6% of the total bacterial cells in the duodenum^61^.

Fungi constitute a significant component of the plant microbiome. Among the four metagenomes analyzed here, dried figs showed the highest number of 18S rRNA sequence reads mapping to *Aspergillus* detected at relatively high frequency. The presence of this fungus and other filamentous species has previously been described in dried figs and their enrichment in the fruit has been linked to environmental conditions such as humidity and temperature during the drying, post-harvest, and manufacturing processes that favor fungal growth^62^. Some of these fungi can produce mycotoxins, including aflatoxin B1, which has carcinogenic effects^63^, but the limited sequencing depth prevents us from assessing whether mycotoxins are present in the metagenome.

SCFAs are the most widely studied microbial metabolites because of their relation to human health. In comparison with the other metagenomes, green olives showed the highest abundance of enzymes participating in lactate, propionate, and acetate production. The production of SCFAs has been experimentally detected in the Moresca and Kalamata cultivars^59^ and in the Spanish-style green table olives, Gordal and Manzanilla olive cultivars^28,64^. Lactate is produced during fermentation by LAB, which was the most abundant taxa in the sample. Moreover, *Acidipropionibacterium* has been positively correlated with propionic acid, acetic acid, and methyl propanoate, while *Ruminococcus* with butyric and propionic acids^28^. Although *Ruminococcus* was not detected in the sample as it is a gut commensal, there are likely other microbial members that could be participating in the production of butyric acid but are present in low abundance or absent in fresh fruits and vegetables.

Vegetables, fruits, and grains are an important source of carbohydrates and dietary fibers in our diet. In particular, dietary fibers are comprised of cell wall polysaccharides that are indigestible to the human host, but are used as fermentable substrates by microbes residing in the large intestine to produce SCFAs^39,65^. There is a large variety of plant cell wall polymers, each with unique chemical bonds that are degraded primarily by specific glycosyl hydrolase (GH) enzyme families. Analysis of the CAZyme families in the metagenomes (Supplementary Table 6) indicated that GH13 and GH23 were the most prevalent families as they are involved in breaking down starch, one of the most abundant and ubiquitous biopolymers derived from plants. In addition, certain families were more frequent in specific samples.

Overall, our study highlights a high microbial diversity in raw fruits and vegetables. Specifically, the role of genomic and functional diversity identified in *Pseudomonas* and *Lactobacillus* needs additional attention, since 100’s million cells are consumed in fresh food items like apples^17^. Moreover, there is a lack of research on studying which part of the edible plant microbiota become possible autochthonous (resident) and/or complementary allochthonous (transient) members of the human gut, and the extent to which these microbes influence, if any, ecological processes such as stability, resilience, and microbial *in situ* evolution.

The large and widespread functional diversity observed in the edible plant microbiome suggests that they may need to be considered in studies of the human gut microbiome, especially when differentiating microbiome-mediated clinical outcomes and in particular during dietary interventions that contain them. This preliminary study warrants further investigation with larger sample size and samples from different sources to evaluate activity and colonization along the many sub-environments of the GI tract and related host effects in health and disease.

## Methods

### Sample collection and microbial isolation

A set of commonly consumed fruits and vegetables (Supplementary Table 1) was purchased in 2017 in a farmers’ market or in a supermarket in San Francisco, California to represent the diversity at the point of sale. The plant materials used in this study were farmed and sold in the United States. We have purchased them at the point of sale and are in compliance with international laws, protocols, and regulations for the Convention of Biological Diversity. Every sample was processed in the same way as it is typically done for consumption, consisting of rinsing with tap water vigorously and blotting dry. For every sample, 50 g were weighed and blended in a kitchen-grade blender (Ninja) for 30 s with 200 ml of a homogenizing buffer containing phosphate-buffered saline (PBS) pH 7.2 and 1% Triton X and 2% 0.5 M EDTA pH 8.0 (10 mM final concentration EDTA). After blending, the slurry was filtered with a coarse and fine kitchen grade strainer and a 40 mM strainer (Cole-Parmer 410-0001-FLS-CP Cell Strainer) sequentially. The filtered suspension was spun down at 5000 rpm for 10 min at 4 °C to pellet down the bacterial and fungal cells and resuspended in PBS for DNA extraction.

To quantify the total bacterial load in the samples, 50 g of sample material was weighed out into a blender cup previously sterilized with 70% EtOH. 200 mL of sterile PBS was added to the blender cup and the sample was blended for 90 s before being strained through a Miracloth (Available from Millipore Sigma 475855). The filtrate was centrifuged for 10 min at 5000 rpm. The supernatant was aspirated. To obtain representative colonies, pelleted material was resuspended in 5 mL of PBS and diluted from 10^0^ to 10^−7^. The filtered suspension was plated on a variety of culture media including standard recipes of R2A, MRS, PDA and TSA agar plates under aerobic or anaerobic conditions, different pH, and temperature. Single colonies were inoculated in liquid medium for 2 days and used for DNA extraction.

### DNA extraction and sequencing of metagenomes and whole genomes

To extract DNA from individual colonies, the Zymo quick DNA bacterial/fungal kit (Zymo) was used. DNA quality and concentration were measured using Nanodrop and Picogreen fluorescent quantification. Genomic libraries were built using the Nextera DNA library preparation kit (Illumina Inc) and sequencing was performed using an Illumina MiSeq instrument (Illumina Inc) with 2 × 250 bp paired-end reads. Alternatively, other bacterial genomes were sequenced using an Oxford Nanopore and their genomes were assembled using flye v2.8.3^66^.

To extract DNA from the microbial pellets obtained from fresh fruits and vegetables, the MagaZorb DNA extraction kit (Promega) was used. DNA quality and concentration were measured using Nanodrop and Picogreen fluorescent quantification. DNA libraries were built using the Nextera XT library preparation kit (Illumina Inc) and DNA sequencing was performed using an Illumina HiSeqX instrument (Illumina Inc) using a 2 × 150 bp flow cell.

### Whole-genome assembly and shotgun metagenomic analyses

Raw reads from genome sequences were filtered for quality control using Solexa QA (Cox et al. 2010) with a Phred score > 20 and a minimum length of 50 bp. Trimmed reads were assembled de novo using IDBA-UD v1.1.3 with pre-corrections and default parameters^67^. Contigs smaller than 1 Kb were filtered from the assembly. Genome completeness and contamination were evaluated using CheckM v1.07^68^.

Raw paired-end reads from the metagenomes were processed for quality control with Solexa QA^69^ for trimming and removing of Illumina adaptors using a Phred score > 20 and minimum fragment length of 50 bp. Host DNA removal from the metagenomes was performed by mapping trimmed reads to each plant reference genome using Bowtie2^70^ with default parameters. Nonpareil v3.0^23^ with default parameters was used to estimate the sequencing average coverage and diversity (similar to Shannon index) for each sequenced and filtered library.

De novo metagenomic assembly, recovery of metagenome-assembled genomes (MAGs), and MAG quality control were performed as described previously^57^. Briefly, assembly was performed using MEGAHIT v1.1.1 with default parameters^71^, binning analysis using MetaBAT2 v2.12.1; option ‘-m 1500’^72^, and estimation of the completeness and contamination of each MAG using CheckM v1.07 with lineage specific workflow^68^. Fragment recruitment plots for bacterial isolates and MAG contigs were constructed using the enveomics.R package v1.4.1 from the Enveomics collection^73^.

Taxonomic annotation of MAGs was performed by identifying the closest relative genome using reference genomes available at RefSeq from NCBI as of November 2019 and mash distances using Mash v2; option ‘‘-s 10000’’ for sketching^74^. Then, the Average Nucleotide Identity (ANI) values were computed between each pair in order to define at which taxonomic level the MAGs belong to using the species (> 95% ANI) and genus (> 80% ANI) scores accordingly^26^. Taxonomic classification of metagenomic reads was carried out using Kraken2 with default parameters^24^.

Phylogenetic analysis of 26 *Pseudomonas* species present in the metagenomic samples with more than 0.1% relative abundance was conducted by using reference genomes available at NCBI and based on 100 universal single copy marker genes from each genome. Identification of single copy genes was performed using the script HMM.essential.rb^73^ and HMMer 3.0^75^, then aligned using muscle v3.8^76^ and concatenated using the script Aln.cat.rb^73^. A maximum-likelihood tree was constructed with RAxML 8.2.12^77^ using the GTRGAMMA model and 100 bootstraps. The best scoring tree was visualized and edited using iTOL v6 (https://itol.embl.de/)^27^.

Protein-coding genes in assembled contigs were identified by Prodigal^78^ with default parameters. Functional annotation was performed using BLASTp^79^ searches of amino acid sequences against the UniProt/SwissProt database^36^ using a cut-off for a match of at least 40% amino acid identity and 70% of the query protein length. The abundance of protein functions in each dataset was calculated as reads per kilobase per million mapped reads (RPKM). Proteins were assigned to the functional categories using Gene Ontology terms^80^. Functional Mapping and Analysis Pipeline^81^ was applied to estimate gene family abundance values associated with metabolic pathways and operons of interest using KEGG Orthologous groups (KOs) and the KEGG enzyme database^82^. Prediction of carbohydrate-active enzymes (CAZymes) in the metagenomes using run-dbcan v2.0.6^83^ and the dbCAN CAZyme database^38^.

All metagenomic samples were deposited in the NCBI’s Sequence Read Archive under the project PRJNA635436.

## Supplementary Information

Supplementary Information.

Supplementary Tables.
