# Supplementary material for: The Edible Plant Microbiome represents a diverse genetic reservoir with functional potential in the human host
Source: Sci Rep. 2021 Dec 15;11:24017. doi: 10.1038/s41598-021-03334-4 (PMC8674285; doi:10.1038/s41598-021-03334-4)
Supplement: Supplementary file 1 — Supplementary Information. [file 41598_2021_3334_MOESM1_ESM.docx]

**The Edible Plant Microbiome represents a diverse genetic reservoir with functional potential in the human host**

Maria J. Soto-Giron, Ji-Nu Kim, Eric Schott, Claudine Tahmin, Thomas Ishoey, Tracy J. Mincer, Jillian DeWalt, Gerardo Toledo

**Description of Supplementary Tables and Figures**

**Supplementary Table 1.** General statistics of metagenomes for each sample including sampling collection date and location, percentage of trimmed sequencing reads, host reads and assembly statistics.

**Supplementary Table 2.** Metadata associated with metagenomes from different environments reported in previous studies^79–82^ .

**Supplementary Table 3.** Metaxa classification. Percentage of sequencing reads recovered as part of ribosomal small subunit (12S/16S/18S) sequences from prokaryotes and eukaryotes in the metagenomes

**Supplementary Table 4.** Binning statistics of metagenome-assembled genome (MAG) sequences recovered from the samples. Taxonomic classification is based on the closest reference genome identified using the Average Nucleotide Identity (ANI) against genomes from RefSeq database at NCBI.

**Supplementary Table 5.** Functional profile of metagenomes analyzed in this study. Relative abundance (reads per kilobase per million reads, RPKM) of GO biological processes annotated in predicted genes from the metagenomic samples using UniProt DB v2019.

**Supplementary Table 6.** Percentage of predicted genes from the metagenomes identified in each CAZyme family. CAZymes are grouped in four main classes: AA: Auxiliary Activity, CBM: Carbohydrate Binding Module, CE: Carbohydrate Esterase, GH: Glycoside Hydrolase, GT: Glycosyltransferase, PL: Polysaccharide Lyase.

**Supplementary Figure 1. Average coverage of shotgun metagenomes from fruits and vegetables analyzed in this study.** Coverage curves based on the redundancy of metagenomics reads in the sample. The line represents the fitted Nonpareil curve^1^ representing how much of the total DNA in the average sample was covered by sequencing. The dashed line represents the Nonpareil projection curve. The horizontal red dashed lines indicate the 100% and 95% coverage levels.

**Supplementary Figure 2. Fragment recruitment plots of the reference genome of *P. fluorescens* NCTC 10038 against sequencing reads from each sample.** The y-axis in the top left panel corresponds to the average sequencing depth values in logarithm scale of reads that mapped to the genome sequence (x-axis). The y-axis in the bottom left panel corresponds to the percentage nucleotide identity (%) of each mapped read and the x-axis to the position of the read on the genome. The right bottom panel shows the identity histogram of mapped reads (logarithmic scale). The dark blue peak in the histogram (right top panel) corresponds to the average coverage of mapped reads with ≥95% nucleotide identity, while light blue indicates the coverage of sequencing reads with <95% nucleotide identity. A sequence-discrete population is represented by reads with high nucleotide identity (> 95% nucleotide identity, indicates the cut-off for species demarcation) to the reference genome sequence and with even coverage across the reference sequence.

**Supplementary Figure 3. Genomic diversity of LAB MAGs recovered from green olives metagenome.** Recruitment plot showing the mapping of metagenomic reads to the genome sequence of each LAB MAG. The y-axis in the top left panel corresponds to the average sequencing depth values in logarithm scale of reads that mapped to the genome sequence (x-axis). The y-axis in the bottom left panel corresponds to the percentage of nucleotide identity (%) of each mapped read and the x-axis to the position of the read on the genome. The right bottom panel shows the identity histogram of mapped reads (logarithmic scale). The dark blue peak in the histogram (right top panel) corresponds to the average coverage of mapped reads with ≥95% nucleotide identity, while light blue indicates the coverage of sequencing reads with <95% nucleotide identity.

**A.**

**
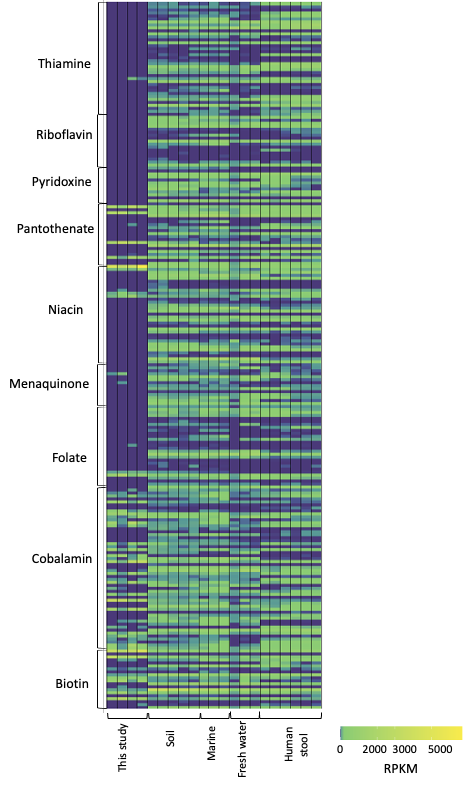
**

**B.**

**Supplementary Figure 4. Metagenomic abundance of microbial-mediated vitamin and short chain fatty acid metabolic pathways in each sample.**  (**A).** Heatmap shows the abundance of KOs associated with the production of short chain fatty acids (**B**). Heatmap showing the abundance of KEGG Orthologies (KOs) expressed in RPKMs that are involved in each vitamin pathway in the samples from this study in addition to other environmental samples. The list of KOs for each metabolic pathway was previously identified in human fecal metagenomes^2^ and probiotic strains (i.e., *Bacillus* *clausii* ENTPro^3^). RPKM was calculated as reads per kilobase per million reads. ​

**References**

1. Rodriguez-R, L. M., Gunturu, S., Tiedje, J. M., Cole, J. R. & Konstantinidis, K. T. Nonpareil 3: Fast Estimation of Metagenomic Coverage and Sequence Diversity. *mSystems* **3**, (2018).

2. Das, P., Babaei, P. & Nielsen, J. Metagenomic analysis of microbe-mediated vitamin metabolism in the human gut microbiome. *BMC Genomics* **20**, 208 (2019).

3. Khatri, I., Sharma, G. & Subramanian, S. Composite genome sequence of *Bacillus clausii*, a probiotic commercially available as Enterogermina®, and insights into its probiotic properties. *BMC Microbiology* **19**, (2019).
